# Physicochemical Insights Into Structuring Olive Oil With Dry‐Fractionated Anhydrous Milk Fat

**DOI:** 10.1111/1750-3841.71493

**Published:** 2026-09-18

**Authors:** Konstantina Zampouni, Prodromos Prodromidis, Elefterios G. Andriotis, Eugenios Katsanidis, Thomas Moschakis

**Affiliations:** ^1^ Department of Food Science and Technology, School of Agriculture, Faculty of Agriculture, Forestry and Natural Environment Aristotle University of Thessaloniki Thessaloniki Greece; ^2^ Faculty of Creative Design and Clothing, School of Design Sciences International Hellenic University Kilkis Greece

## Abstract

Anhydrous milk fat (AMF) and its dry‐fractionated derivatives were evaluated as structurants for olive oil oleogels designed to reduce the overall saturated fat content compared to conventional solid fats. AMF was subjected to sequential dry fractionation at temperatures ranging from 5°C to 30°C to produce liquid and solid fractions differing in fatty acid composition and thermal behavior. The fractions were characterized using GC‐FID, Raman and ATR‐FTIR spectroscopy, polarized and confocal microscopy, rheological measurements, and thermal analysis. Based on their distinct physicochemical properties, the 30°C fractions (S30 and O30) were further investigated as oleogelators in olive oil. The S30 fraction was enriched in saturated fatty acids, exhibited higher melting temperatures and formed dense microstructures composed of numerous fine crystals. Rheological measurements revealed that the S30 generated robust oleogels at significantly lower concentrations than unfractionated AMF, demonstrating high structuring efficiency through the formation of a strong crystalline network. However, these oleogels also exhibited greater brittleness, consistent with their denser crystal packing. Overall, dry‐fractionated AMF effectively structured olive oil into stable oleogels. Despite its higher saturated fatty acid content, the S30 fraction formed self‐supporting oleogels at a structurant concentration of only 25% (w/w). Consequently, oleogels containing 25% S30 and 75% olive oil may provide structured lipid systems with a lower overall saturated fatty acid content than formulations based entirely on AMF or conventional solid fats while maintaining desirable mechanical and functional properties. These findings highlight the potential of dry‐fractionated AMF as a natural, food‐grade oleogelator for developing alternative structured lipid systems.

## 1 Introduction

Milk fat (MF) is a nutritionally and technologically versatile lipid system that contributes to the characteristic flavor and sensory attributes of dairy products (Guo et al. 2025). MF is a highly heterogeneous lipid system composed of a mixture of triacylglycerols (TAGs), which constitute up to 98% of the total lipid content, along with minor components, such as phospholipids (PLs, 1%–2% of total lipids) and free fatty acids (FFAs). More than 400 fatty acids (FAs) and over 200 TAGs have been identified in MF (Liu et al. 2020; Gómez‐Mascaraque et al. 2021; Si et al. 2023), contributing to its broad melting range, ranging from −40°C to 40°C, and complex physicochemical behavior (Si et al. 2023). Consequently, the dairy and food industry has focused on separating natural MF through fractionation to obtain lipid fractions with tailored melting profiles suitable for diverse food applications (Iung et al. 2025; Si et al. 2023). Although both chemical and physical modification techniques have been investigated (Mathiasen et al. 2024), chemical approaches remain limited in commercial use due to safety, cost, and regulatory constraints. In contrast, physical technologies, including dry fractionation, distillation, ultrasonication, high‐pressure processing, and supercritical fluid extraction, are more extensively applied. Dry fractionation is the most commonly used industrial method for producing commercial fat fractions, due to its simplicity, low energy requirements, and solvent‐free processing (Mathiasen et al. 2024; Mohan et al. 2021; Si et al. 2023).

Anhydrous milk fat (AMF) contains more than 99.5% MF with minimal moisture and non‐fat solids (Elbarbary et al. 2025). Its relatively high melting range (32°C–36°C) limits its use in products requiring softer textures (Gómez‐Mascaraque et al. 2021), although it provides desirable dairy aroma and flavor due to the short‐chain saturated fatty acids (SC‐SFAs), mouthfeel, and good thermal and storage stability. MF is typically fractionated into three high melting point fractions (HMPF), medium melting point fractions (MMPF), and low melting point fractions (LMPF) via controlled crystallization. Fractionation not only enables the production of lipid fractions with specific functional properties but also is a critical step in the manufacture of butter, fresh cream, and ice cream (Si et al. 2023). MF exhibits polymorphic behavior of fats rich in long‐chain SFAs, crystallizing into α‐, β‐, and β‐forms depending on cooling conditions. The α‐crystals are formed at ∼10°C–20°C, while β′‐crystals are developed at temperatures below 10°C. Transition to the thermodynamically stable β‐crystals is time‐dependent and therefore lacks a single defining temperature (Mohan et al. 2021). Notably, MF differs from many other fats in the relative stability of the β′polymorphs, as β‐crystals remain detectable even after prolonged storage (Mortensen 2016). For example, the α‐crystalline form melts below 18°C and recrystallizes into the β′ polymorph, which melts between 16°C–25°C, while the more stable β form melts at higher temperatures (∼25°C–36°C) (Mohan et al. 2021). These polymorphic transitions strongly influence the functionality of fat‐based systems. Current dietary policy and nutritional guidance have increased interest in lipid systems with reduced saturated fatty acids (SFAs) and trans fatty acids (TFAs). The World Health Organisation (WHO) recommends limiting total fat intake to < 30% of total energy, with SFAs contributing < 10% and TFAs < 1% (Pușcaș et al. 2020). In this context, the replacement of solid fats, such as palm and coconut oils, has become a priority, motivating the development of structured lipid systems that meet both functional and nutritional criteria (Bascuas et al. 2021; Pușcaș et al. 2020; Syan et al. 2024).

Oleogelation has emerged as a promising fat structuring technique, converting liquid oils into semi‐solid three‐dimensional networks, using several approaches (Q. Wang et al. 2025) thereby enabling substantial reductions in SFA content while preserving desired textural properties (Farooq et al. 2025; Gómez‐Mascaraque et al. 2021). Several edible oils have been successfully structured into oleogels (Dimakopoulou‐Papazoglou et al. 2023; Prodromidis et al. 2023; Xu et al. 2023; Zampouni, Sideris, et al. 2024; Zampouni, Soniadis, Moschakis, et al. 2022), including olive oil, a dominant component of the Mediterranean diet. Olive oil is characterized by a high oleic acid content (∼70%–75% of total FAs) and bioactive constituents (sterols, polyphenols) associated with antioxidant and anti‐inflammatory effects (Tsimihodimos and Psoma 2024). Oleogels have been used as an alternative to conventional fats, reducing the SFA content while maintaining sensory characteristics of the produced foods (Guo et al. 2023; Salama et al. 2025; Zampouni, Soniadis, Dimakopoulou‐Papazoglou, et al. 2022). Moreover, oleogels have functioned as delivery systems for bioactive compounds (Andriotis et al. 2022, 2024; Farooq et al. 2024; Kanelaki et al. 2022; Xu et al. 2023) or as lipid phases in the formation of bigel systems. Therefore, oleogelation provides a useful approach for investigating how variations in the composition and crystallization behavior of AMF fractions influence their oil‐structuring functionality. Although such oleogel systems have previously been investigated, limited information is available regarding the use of sequentially dry‐fractionated AMF fractions as tailored oleogelators with distinct physicochemical characteristics and structuring efficiencies. In particular, the influence of dry fractionation on the crystallization behavior, thermal properties, and oleogelation performance of individual AMF fractions remains insufficiently explored.

Therefore, the aim of this study was to evaluate the structuring ability of AMF and its dry‐fractionated solid and liquid fractions to structure olive oil into oleogels. AMF and its fractions were first characterized, followed by the evaluation of the thermal, microstructural, and rheological properties of the resulting oleogels. The overall objective was to assess the feasibility of using dry‐fractionated AMF as natural, food‐grade structurants for the development of healthier fat formulations.

## 2 Materials and Methods

### 2.1 Dry Fractionation Process

**FIGURE 1:**
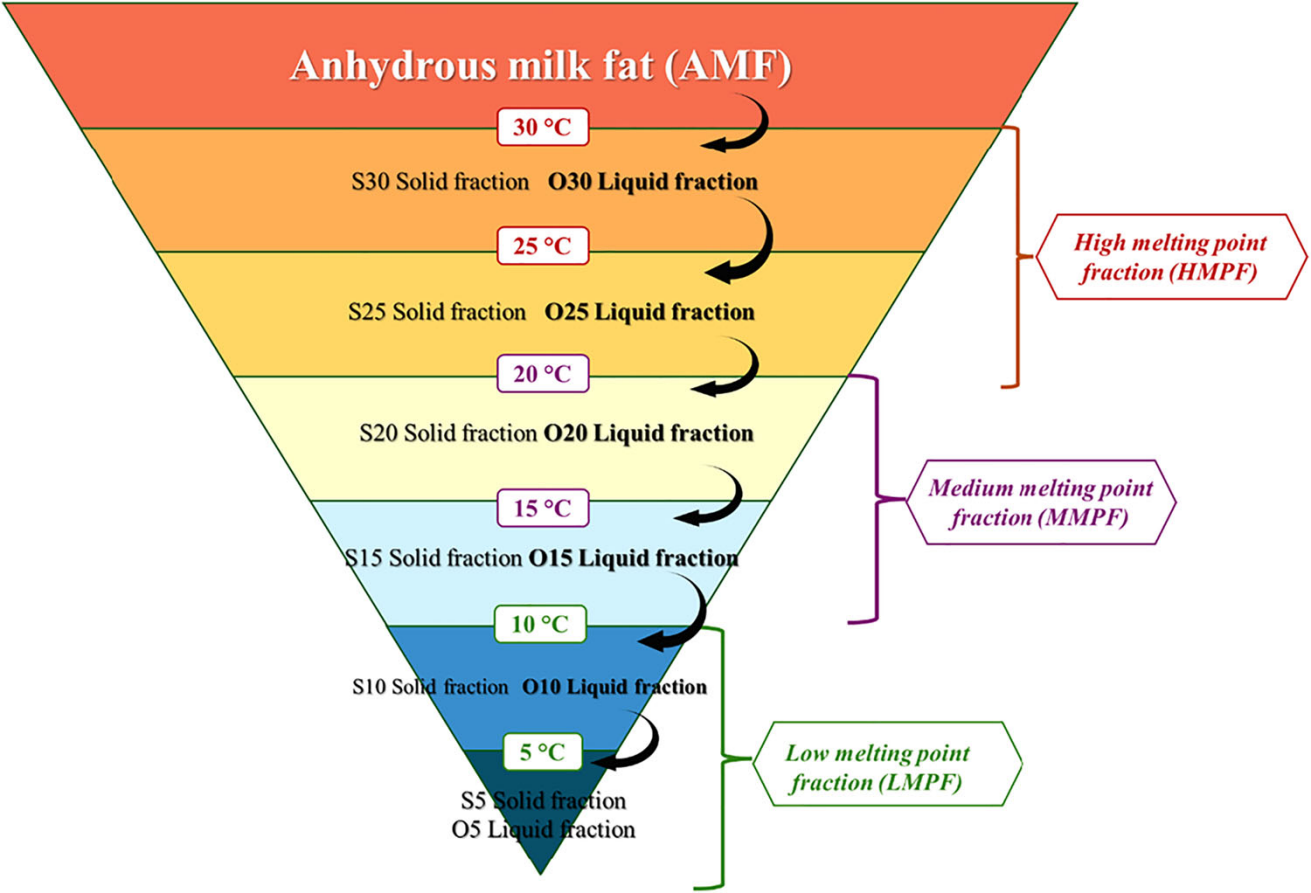
Schematic representation of the dry fractionation process of anhydrous milk fat (AMF) at different temperatures. Sequential cooling at 30°C, 25°C, 20°C, 15°C, 10°C, and 5°C corresponding solid (S30–S5) and liquid (O30–O5) fractions. These fractions are grouped according to their melting characteristics into high melting point fractions (HMPF), medium melting point fractions (MMPF), and low melting point fractions (LMPF).

The dry fractionation method was adapted for AMF (EPIRUS S.A., Arta, Greece) according to the literature with slight modifications (Si et al. 2023). Briefly, 100 g of AMF were melted by heating at 60°C under continuous magnetic stirring (150 rpm). The molten AMF was maintained at 60°C for 30 min to eliminate any previous crystalline memory and then incubated at 30°C in an incubator (Friocell, MMM Medcenter Einrichtungen GmbH, Munich, Germany) for 24 h to induce partial crystallization. After incubation, the samples were centrifuged (Gyrozen 1248R High‐speed Centrifuge, Gimpo, South Korea) for 40 min at 10,000 × *g* at 30°C. Then, the liquid fraction was collected and subjected to the same process (incubation at 25°C for 24 h and centrifugation for 40 min at 10,000 × *g* at 25°C). This sequential process was repeated for incubation temperatures of 20°C, 15°C, 10°C, and 5°C. The fractionation yield was determined gravimetrically. The described process was also applied to all collected solid fractions to ensure no further phase separation occurred. The selected fractionation temperatures were chosen to progressively separate TAG fractions with different melting and crystallization characteristics, thereby formulating AMF fractions with distinct compositional, thermal, and structural properties. The dry fractionation procedure was performed in triplicate and the process sequence is illustrated in Figure 1.

### 2.2 Determination of FAs

The determination of FAs in AMF and its fractions was performed using Gas Chromatography—Flame Ionization Detector (GC‐FID) analysis. Briefly, 0.1 g of MF was weighed and placed into a test tube. Then, 2 mL of hexane (ChemLab, Zedelgem, Belgium) and 0.2 mL of a methanolic solution (0.1 M) of potassium hydroxide were added to methylate the FAsto form fatty acid methyl esters (FAMEs). The mixture was stirred for 1 min and allowed to equilibrate until the supernatant phase containing the FAMEs became transparent. The supernatant phase was collected, filtered (0.45 µm PTFE hydrophobic filters) and analyzed using a gas chromatography system (TRACE GC 2000 Series, Thermo Quest CE Instruments, Wigan, UK) equipped with a FID and an automatic sampler (TRIPLUS AS Thermo Quest CE Instruments). The FAMEs were analyzed using a BPX70 GC column (30 m in length, 0.32 mm in diameter, and 0.25 µm in thickness, SGE Analytical Science). Helium was used as a carrier gas with a continuous flow of 2 mL/min. The injection port and detector temperatures were set at 250°C, with a split ratio of 1:20. Initially, the column oven temperature was set at 46°C for 2 min, then increased to 130°C at a rate of 50°C/min for 10 min. Subsequently, it was raised to 175°C at 2°C/min and maintained at 175°C for 2 min. The temperature then increased to 200°C at 3°C/min and held at 200°C for 3.5 min before reaching 240°C at a rate of 10°C/min, where it was allowed for 5 min. The total measurement duration was 60 min. No unresolved or late‐eluting peaks were observed under the selected chromatographic conditions. Detection and identification of FAMEs were performed by comparing sample retention times with those of a certified 37‐component FAME reference standard (AccuStandard, New Haven, CT, USA), analyzed under identical chromatographic conditions. The standard mixture contained methyl esters of C4:0, C6:0, C8:0, C10:0, C11:0, C12:0, C13:0, C14:0, C14:1, C15:0, C15:1, C16:0, C16:1, C17:0, C17:1, C18:0, C18:1 *trans*, C18:1 *cis*, C18:2 *trans*, C18:2 *cis*, C18:3, C18:3 n‐3, C20:0, C20:1, C20:2, C20:3, C20:3 n‐3, C20:4, C21:0, C22:0, C22:1, C22:2, C23:0, C24:0, C24:1, C20:5 and C22:6. The data were processed using Chrom Quest 5.0 software (ver. 3.2.1, Thermo Separation Products). The parameters used to characterize the FAs profile included the total SFAs, monounsaturated fatty acids (MUFAs), polyunsaturated fatty acids (PUFAs), the ratio of saturated to unsaturated fatty acids (SFA/UFA), SC‐SFAs (C4:0), medium‐chain saturated fatty acids (C6:0–C12:0) (MC‐SFAs), and long‐chain saturated fatty acids (> C13:0) (LC‐SFAs).

### 2.3 Preparation of Oleogels

AMF and its fractions were utilized as oleogelators to structure olive oil (Minerva S.A., Metamorphosi, Greece). In brief, predetermined quantities of refined olive oil and selected structuring agents, such as AMF, S30, and O30, were added in various concentrations in oil, ranging from 50 to 10 (% w/w). The reported concentrations correspond to the proportion of AMF or AMF fraction relative to the total weight of the oleogel system. Preliminary evaluation of all fractions indicated that S30 and O30 exhibited the most distinct crystallization and melting behavior among the obtained fractions and were therefore selected for further oleogelation studies. The mixtures were heated to 60°C under continuous magnetic stirring until the structurants were fully solubilized. To eliminate any previous thermal history, the solutions were maintained at 60°C for an additional 30 min. The mixtures were then subjected to two cooling conditions, representing slow and fast cooling, by setting them at 20°C or at rapid cooling in an ice bath at 0°C for 1 h, respectively. Βoth conditions were initially evaluated; however, only oleogels prepared under rapid cooling conditions formed stable networks. Therefore, all samples used for further analyses were prepared under rapid cooling conditions (0°C). Pure olive oil, used as the continuous phase, did not form self‐standing gel structures under the conditions applied in this study.

### 2.4 Microscopy Analysis

#### 2.4.1 Polarized Light Microscopy

Micrographs of AMF, its fractions and the formed oleogels were captured using polarized light microscopy (PLM). An Olympus BX43 microscope (Olympus Optical Co. Ltd., Tokyo, Japan) equipped with a digital camera (Basler USB3 Vision, Germany) and a 20× objective lens (numerical aperture of 0.75) was utilized. This magnification was considered appropriate for comparative evaluation of the microstructural organization of the different oleogel systems. Samples were placed on glass microscopy slides and gently covered with coverslips to form a thin layer that enabled improved observation at predetermined intervals. Specifically, the oleogels were evaluated after 7 days. To prevent moisture loss, a thin protective varnish coating was applied around the coverslip edges. All analyses were performed at ambient temperature.

#### 2.4.2 Confocal Laser Scanning Microscopy

Confocal laser scanning microscopy (CLSM) was employed to evaluate the microstructure of the oleogels prepared with AMF and its fractions. Nile blue (0.01 g/100 g) and Nile Red (0.01 g/100 g) fluorescent dyes were used to stain the crystalline and lipid phases, respectively. The samples were placed in specialized trough slides (0.15 mm thickness) from WillCo Wells BV (Amsterdam, the Netherlands) and examined under confocal microscopy at predetermined intervals. The microstructure was analyzed using a Leica TCS SP5 II confocal scanning laser microscope (Leica Microsystems GmbH, Wetzlar, Germany) mounted on a Leica Model DM 6000B inverted microscope operating under fluorescence. An immersion objective lens with 63× magnification and a numerical aperture of 1.40 was used. Images were acquired approximately 20–30 µm beneath the coverslip to minimize hydrodynamic interactions. Fluorescence excitation was achieved using a 488 nm argon laser for Nile Red and a 633 nm red HeNe laser for Nile blue. To minimize channel cross‐talk, Nile red and Nile blue fluorescence signals were acquired sequentially using their respective excitation wavelengths. The resulting images showed clear spatial separation between the lipid and crystalline phases, with no evident channel overlap under the acquisition conditions used. The same acquisition settings were used for all comparative images. Micrographs were collected at 512×512 pixels in the *x*–*y* plane, with each image averaged over eight scans. All measurements were performed at ambient temperature.

### 2.5 Thermal Analysis

The thermal behavior of AMF fractions and the corresponding oleogels during melting and crystallization was analyzed using a micro–differential scanning calorimeter (microCalvet μDSC 7 Evo‐1A, Setaram, Caluire‐et‐Cuire, France), equipped with two parallelly positioned 1 mL capsules. One DSC pan contained the sample, while the other contained olive oil as the reference. Using olive oil as the reference for both the pure AMF fractions and the corresponding oleogel systems ensured a consistent baseline throughout all measurements and enabled direct comparison of the thermal behavior of the pure fractions with that of the corresponding olive‐oil‐based oleogel systems. The thermal protocol consisted of four phases. In the first phase (fast heating), the temperature increased from 20°C to 30°C. This preliminary heating step was included to partially melt low‐melting crystals formed during storage at room temperature and to standardize the initial thermal state of the samples before the main heating scan. In the second phase (1st heating), the temperature was further raised from 30°C to 60°C. This was followed by the third phase (cooling), during which the temperature was lowered from 60°C to −10°C. In the final phase (2nd heating), the temperature increased again from −10°C to 60°C. Heating and cooling rates were set at ± 1°C/min and the sample mass was 400 ± 10 mg. Measurements were conducted under a continuous flow of nitrogen gas (0.8 bar) to maintain an inert environment. The μDSC was calibrated using a standard sample of pure naphthalene. Onset crystallization temperature (*T*_c_), onset melting point (*T*_m_), and offset melting point were calculated using CALISTO software (Setaram, Caluire‐et‐Cuire, France).

### 2.6 Rheological Studies

The rheological properties of AMF fractions and the corresponding oleogels were analyzed using a Physica MCR 302 rotational rheometer (Physica Messtechnik GmbH, Stuttgart, Germany). Temperature regulation was achieved using a Paar Physica water bath and a Peltier system (TEZ 150P/MCR) with an accuracy of ± 1°C. Rheological data were analyzed with Rheoplus V2.21 software (Anton Paar GmbH, Austria). All samples were studied using parallel plate geometry (PP50 sandblasted, with a diameter of 50 mm and a 1 mm gap). Initially, strain sweep experiments at a frequency of 1 Hz were performed to determine the linear viscoelastic region (LVR). A strain of 0.01% was selected to ensure measurements were conducted within the LVR and to avoid disruption of the crystalline network structure. The viscoelastic properties, including the elastic modulus (*G*′), viscous modulus (*G*″) and the tangent of the phase angle *δ* (tan*δ* = *G*″/*G*′), were recorded during frequency sweeps within the range of 0.1–100 Hz. The apparent yield stress (*τ*_γ_) was determined as the shear stress value at which the *G*′ value deviates by 10% from the LVR during strain sweep (Moschakis et al. 2016). The flow point was defined as the *G*′–*G*″ crossover point (tan*δ* = 1) during the strain sweep test and was expressed as the corresponding shear stress (Pa) (Sein et al. 2002; Prodromidis et al. 2023, 2024). All measurements were performed in triplicate (three samples per formulation), with each sample measured in duplicate. All measurements were conducted at 20°C. Prior to rheological measurements, all samples (AMF and its fractions) were prepared and allowed to crystallize, then the samples were carefully loaded onto the rheometer to avoid disruption of the crystalline network.

### 2.7 ATR‐FTIR Analysis

ATR‐FTIR spectra of AMF, its fractions and the resulting olive oil oleogels were recorded using a Fourier transform infrared spectrometer (FTIR 6700 series, JASCO, Tokyo, Japan) equipped with a three‐reflection diamond ATR unit (MIRacle ATR, Pike Technologies, Madison, USA). Spectra were collected in the range of 600–4000 cm^−1^ with 32 scans at a resolution of 4 cm^−1^. Before each measurement, an open‐air background spectrum was recorded as a blank and five spectra were obtained for each sample. All the tests were carried out at ambient temperature. The original spectra were corrected for baseline, water vapor, CO_2_ and ATR noise corrections using the Spectra Manager V.2 software (JASCO, Tokyo, Japan). All spectral data are presented as transmittance (%).

### 2.8 Raman Spectroscopy

The Raman spectra of fractionated AMF were obtained using a Confocal Raman microscope (XploRA Plus, Horiba Instruments Inc., Kyoto, Japan). The system consisted of an Olympus BX41 microscope, Xplora Raman imaging spectrometer, and a motorized three‐dimensional microscope stage (XYZ‐dimension). Spectrum analysis was performed using Horiba LabSpec6 software (Kyoto, Japan). A diode laser (785 nm, 100 mW) was used as the excitation source. Spectra were captured with a 100× objective lens (MPlanFL N, Olympus, Germany) with a numerical aperture of 0.9, covering the range of 400–3900 cm^−1^, using a 600 grooves/mm grating, a 100 µm slit, and a confocal aperture of 300 µm. The acquisition time for the spectra was 10 s, with five accumulations. Samples were placed on an objective holder plate and positioned on the microscope stage for spectrum acquisition. All measurements were conducted at ambient temperature.

### 2.9 Statistical Analysis

Statistical comparisons were performed separately within each experimental dataset using one‐way analysis of variance (ANOVA). Differences between the samples were identified using Duncan's multiple range test, calculated with SPSS Statistics 25 (SPSS Inc., Chicago, IL, USA), at a significance level of *p* < 0.05. All values are presented as the means of at least three independent replicates.

## 3 Results and Discussion

### 3.1 Characterization of AMF Fractions

#### 3.1.1 Dry Fractionation Process

Τhe sequential dry fractionation of AMF yielded solid fractions at each crystallization temperature, reflecting the heterogeneous distribution of TAGs in MF. As shown in Table 1, AMF was primarily composed of high‐melting TAGs, with approximately 39% of the total material crystallizing at temperatures above 20°C. A similarly high proportion (∼40%) crystallized within the intermediate range of 10°C–20°C. The dominance of these higher temperature AMF fractions is consistent with the TAGs profile of AMF, which is enriched in long‐chain SFAs and MUFAs. Fractions with low melting temperatures (≤ 10°C) accounted for about 21% of the total mass, including roughly 10% crystallizing below 5°C, indicating that AMF contains a low proportion of short‐chain, medium‐chain, or highly unsaturated TAGs. The yield distribution highlights the compositional heterogeneity of AMF and demonstrates the effectiveness of dry fractionation as a method to segregate TAGs according to their melting behavior. Although the S30 fraction exhibited the highest structuring efficiency, as discussed in Section 3.2.1, its relatively low recovery yield (6%) may represent a limitation for large‐scale industrial application. Therefore, further optimization of the dry fractionation process would be necessary to enhance the recovery of high‐melting fractions while preserving their desirable structuring functionality.

**TABLE 1:** Yield (%) and cumulative yield (%) obtained during sequential dry fractionation of anhydrous milk fat (AMF) at decreasing crystallization temperatures (30°C, 25°C, 20°C, 15°C, 10°C, 5°C, and < 5°C).

Fractionation temperature	Yield (%)	Cumulative yield (%)
30°C	6.0 ± 0.2	6.0
25°C	32.9 ± 1.0	38.9
20°C	10.4 ± 0.3	49.3
15°C	29.4 ± 1.0	78.7
10°C	8.7 ± 0.2	87.4
5°C	2.3 ± 0.1	89.7
< 5°C	10.3 ± 0.2	100.0

#### 3.1.2 Determination of the FA Composition

The FA profiles of AMF and its fractions obtained by dry fractionation at various temperatures are presented in Table 2. The S30 fraction (solid fraction at 30°C) exhibited the highest proportion of SFAs among all fractions, with myristic (C14:0), stearic (C18:0), and palmitic (C16:0) acids constituting the major components. Despite its high SFAs content, the S30 still retained a considerable proportion of UFAs, including oleic acid (C18:1 *cis*‐ω9) and other nutritionally favorable FAs. Their final concentration was around 17.2% (with total UFAs at 20.1%). This level is relatively high, considering the elevated melting point of the S30 fraction. In addition, as the fractionation temperature decreased, the percentage of UFAs increased relative to the 30°C solid fractions. In contrast, the corresponding liquid fractions (O) contained more unsaturated (mono‐ and polyunsaturated) FAs compared to their respective solid counterparts. A temperature‐dependent redistribution of MC‐SFAs and LC‐SFAs was also observed, with a lower fractionation temperature resulting in higher MC‐SFAs and lower LC‐SFAs contents. Dry fractionation of AMF resulted in moderate but functionally significant changes in FAs composition, particularly in the SFA/UFA ratio, which influenced the thermal and structuring behavior of the fractions. It should be noted that dry fractionation operates by selectively crystallizing intact TAG species according to their melting profiles, without hydrolyzing or chemically modifying them. Consequently, the redistribution of TAGs between solid and liquid fractions leads to pronounced differences in physical properties, while only minor changes are observed in the overall FA composition. Similar findings have been reported in previous studies, indicating that a decrease in the SFAs to UFAs ratio has been associated with reductions in crystallization temperature (Gómez‐Mascaraque et al. 2021; Y. Wang et al. 2019). Moreover, butyric acid (SC‐SFAs), which contributes to the characteristic aroma of dairy fat, was primarily found in high‐temperature solid fractions. A higher relative proportion of C18:2 cis‐ω6 (linoleic acid) was observed in the liquid fractions compared to AMF. The high levels of SFAs in the S15 fraction compared to the S20 fraction may account for the increased fractionation yield observed at 15°C (Table 1).

**TABLE 2:** Average fatty acid composition (% w/w) of anhydrous milk fat (AMF) and its fractions obtained by sequential dry fractionation at different crystallization temperatures. AMF fractions follow the convention used in Figure 1.

	AMF fractions								
Fatty acids	AMF	S30	S25	S20	S15	S10	S5	O30	O5
**Butyric acid (C4:0)**	3.8	5.3	4.5	4.3	3.4	3.4	1.6	3.2	3.5
**Caproic acid (C6:0)**	2.9	2.8	2.7	3.1	2.9	3.2	3.2	2.7	3.2
**Caprylic acid (C8:0)**	2.7	2.5	2.4	2.7	2.8	3.0	3.1	2.6	3.1
**Capric acid (C10:0)**	8.1	5.0	3.9	2.7	8.4	9.4	8.7	8.0	8.7
**Undecanoic acid (C11:0)**	0.2	0.3	0.0	0.0	0.0	0.4	0.3	0.2	0.3
**Lauric acid (C12:0)**	4.3	5.4	5.6	5.0	5.1	4.8	4.7	4.4	4.7
**Myristic acid (C14:0)**	12.0	12.6	11.5	10.9	10.4	10.9	11.0	12.0	10.6
**Myristoleic acid (C14:1 *cis‐*9)**	0.0	0.0	0.0	0.0	0.0	0.7	0.7	0.3	0.5
**Pentadecanoic acid (C15:0)**	1.0	1.0	1.2	1.2	1.1	0.4	0.4	0.3	0.4
**Pentadecenoic acid (C15:1 *cis‐*10)**	0.0	0.0	0.0	0.0	0.0	0.1	0.9	1.0	0.9
**Palmitic acid (C16:0)**	29.2	30.9	30.8	30.2	27.3	25.0	25.2	26.6	23.0
**Palmitoleic acid (C16:1 *cis*)**	0.8	1.0	0.9	1.2	1.0	1.3	1.3	0.9	1.3
**Heptadecanoic acid (C17:0)**	0.5	0.5	0.4	0.5	0.7	0.4	0.4	0.6	0.4
**Heptadecenoic acid (C17:1 *cis*‐10)**	0.0	0.2	0.2	0.2	0.2	0.2	0.0	0.0	0.2
**Stearic acid (C18:0)**	10.4	12.1	12.2	10.7	9.8	8.7	8.7	10.3	8.3
**Elaidic acid (C18:1 *trans*‐ω9)**	0.4	0.0	0.9	0.8	0.7	0.6	0.6	0.7	0.6
**Oleic acid (C18:1 *cis*‐ω9)**	19.0	17.2	17.7	21.6	20.6	22.6	24.4	20.2	24.5
**Linoleic acid (C18:2 *cis‐*ω6)**	1.5	1.2	1.6	2.0	1.7	0.6	0.6	2.5	1.7
**Arachidic acid (C20:0)**	1.9	1.5	2.1	1.3	2.3	3.1	2.8	2.0	2.9
**Eicosenoic acid (C20:1 *cis*‐ω9)**	0.5	0.2	0.5	0.7	0.6	0.6	0.7	0.5	0.7
**Linolenic acid (C18:3 *cis‐*ω3)**	0.9	0.3	1.0	1.0	1.0	0.7	0.6	0.8	0.7
**SFA**	76.9	79.9	77.2	72.6	74.2	72.8	70.2	73.0	67.9
**UFA**	23.2	20.1	22.7	27.4	25.8	27.2	29.8	27.0	31.0
**SFA/UFA**	3.3	4.0	3.4	2.7	2.9	2.7	2.4	2.7	2.2
**MUFA**	20.3	18.6	19.3	23.6	22.4	25.4	27.9	22.9	30.2
**PUFA**	2.4	1.5	2.6	3.0	2.7	1.2	1.2	3.3	2.4
**SC‐SFA (C4:0)**	3.8	5.3	4.5	4.3	3.4	3.4	1.6	3.2	3.5
**MC‐SFA (C6:0 – C12:0)**	18.2	16.0	14.6	13.5	19.2	20.7	20.0	17.9	20.0
**LC‐SFA (C13:0 – C21:0)**	54.9	58.6	58.1	54.8	51.6	48.6	48.5	51.8	44.3

*Note*: Data include individual fatty acids as well as grouped categories: saturated fatty acids (SFA), unsaturated fatty acids (UFA), monounsaturated fatty acids (MUFA), polyunsaturated fatty acids (PUFA), short‐chain SFA (SC‐SFA; C4:0), medium‐chain SFA (MC‐SFA; C6:0–C12:0), and long‐chain SFA (LC‐SFA; >C13:0). Values are expressed as means, with standard deviations below ± 0.3%.

#### 3.1.3 ATR‐FTIR Analysis

**FIGURE 2:**
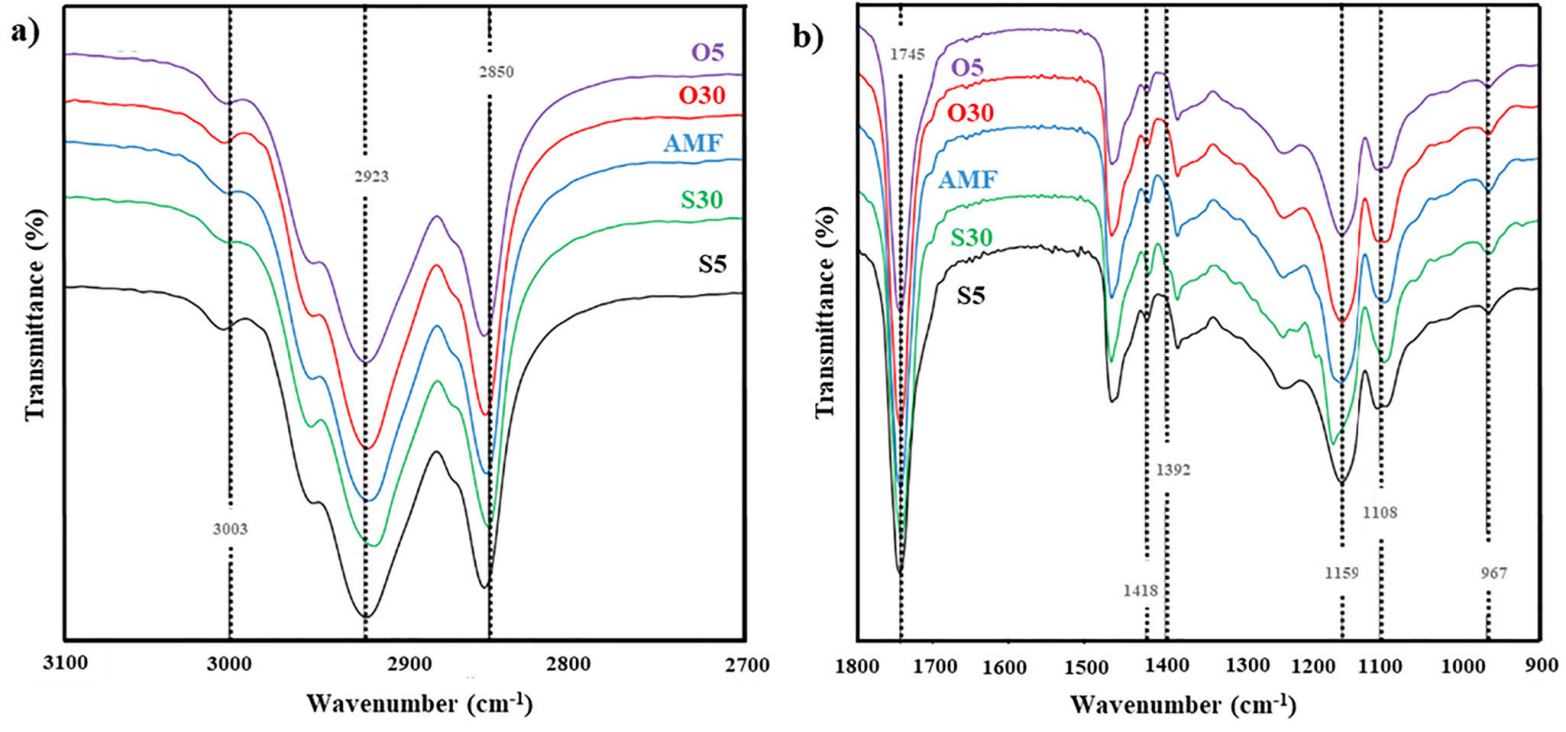
ATR‐FTIR spectra of anhydrous milk fat (AMF) and its dry fractions obtained at two different crystallization temperatures (30°C and 5°C). The fractions include S30 (solid fraction at 30°C), O30 (liquid fraction at 30°C), S5 (solid fraction at 5°C), and O5 (liquid fraction at 5°C). (a) Spectral region from 2700–3100 cm^−1^ and (b) spectral region from 900–1800 cm^−1^.

Analysis of the FA profiles showed that the AMF fractions contained varying proportions of FAs with comparable chain lengths, leading to measurable differences in their degree of unsaturation. According to the literature (Antony et al. 2017), the characteristic vibrational region for edible fats in FTIR spectra is located between 700 and 1390 cm^−1^. As shown in Figure 2, no new peaks were detected in AMF fractions, due to their similar FA composition. However, several notable peak shifts were observed, which may reflect differences in chain length and degree of unsaturation. A characteristic shift of the *cis* double bond stretching vibration was detected from 3003 to 3007 cm^−1^ in the liquid fraction at 30°C (O30) and in both solid and liquid fractions at 5°C (S5 and O5) (Figure 2a). This band corresponds ═C─H stretching in *cis* UFAs (Antony et al. 2017; Talari et al. 2015), indicating an increase in interactions among the UFAs of the fractions, which is in agreement with higher UFAs content (Table 1). Additional shifts were observed in the methylene stretching region. In the S30 fraction, the peak at 2923 cm^−1^ shifted to 2927 cm^−1^ (asymmetric CH_2_ stretching), while the peak at 2854 cm^−1^ shifted to 2850 cm^−1^ (symmetric CH_2_ stretching vibrations). These shifts may indicate differences in chain packing and are consistent with the higher proportion of LC‐SFAs in S30, according to the results from gas chromatography (Table 1). At lower wavenumbers in the carbonyl region (Figure 2b), shifts from 1745 to 1740 cm^−1^ were observed in S30 and the AMF. This band is associated with the C═O stretching of ester groups in TAGs and the number of double bonds between carbon atoms in FAs (Talari et al. 2015). This shift may be associated with fewer interactions in AMF and S30 fractions as they contain fewer UFAs compared to the other fractions. In addition, further changes were detected in the deformation and bending regions. The CH_2_ deformation band, typically observed near 1418 cm^−1^ in UFA‐rich fractions (e.g., O30, O05), was shifted to 1412 cm^−1^ in AMF and S30. This downward shift may reflect the increased chain order and reduced unsaturation, consistent with their higher SFA content. The detection of the peak at 1392 cm^−1^, corresponding to asymmetric CH_2_ bending vibrations (Talari et al. 2015), also is consistent with this interpretation. Also, several wavenumber shifts associated with C─C stretching vibrations were recorded (Figure 2b). Peaks at 1159 cm^−1^ shifted to 1172 cm^−1^, those at 1113 to 1108 cm^−1^, and peaks at 967 cm^−1^ shifted to 961 cm^−1^ in both S30 and AMF samples. These shifts may reflect differences in the chain length distribution and saturation level. All observed differences in the ATR‐FTIR spectra of the AMF fractions are summarized in Table 3. Overall, these spectral variations should be interpreted as qualitative indicators of compositional and structural differences among the AMF fractions.

**TABLE 3:** Summary of wavenumber shifts and peak assignments observed in the ATR‐FTIR spectra of anhydrous milk fat (AMF) and its fractions obtained by dry fractionation at different crystallization temperatures. AMF fractions follow the convention used in Figure 1.

	AMF fractions									Peak assignment
	AMF	S30	S25	S20	S15	S10	S5	O30	O5	
**Wavenumbers (cm^−1^)**	3007	3007	3007	3007	3007	3007	3003	3003	3003	═C─H groups of unsaturated fatty acids
	2923	2927	2923	2923	2923	2923	2923	2923	2923	ν_as_ CH_2_ (asymmetric stretching)
	2854	2854	2854	2854	2854	2854	2850	2850	2850	ν_s_ CH_2_ (symmetric stretching)
	1740	1740	1745	1745	1745	1745	1745	1745	1745	ν (C═O)
	1412	1412	1418	1418	1418	1418	1418	1418	1418	Deformation C─H
	—	1392	—	—	—	—	—	—	—	CH_2_ asymmetric bending, COO‐stretch
	1172	1172	1159	1159	1159	1159	1159	1159	1159	ν (C─C)
	1108	1108	1113	1113	1113	1113	1113	1108	1113	ν (C─C)
	961	961	967	967	967	967	967	967	967	ν (C─C)

#### 3.1.4 Raman Spectroscopy

**FIGURE 3:**
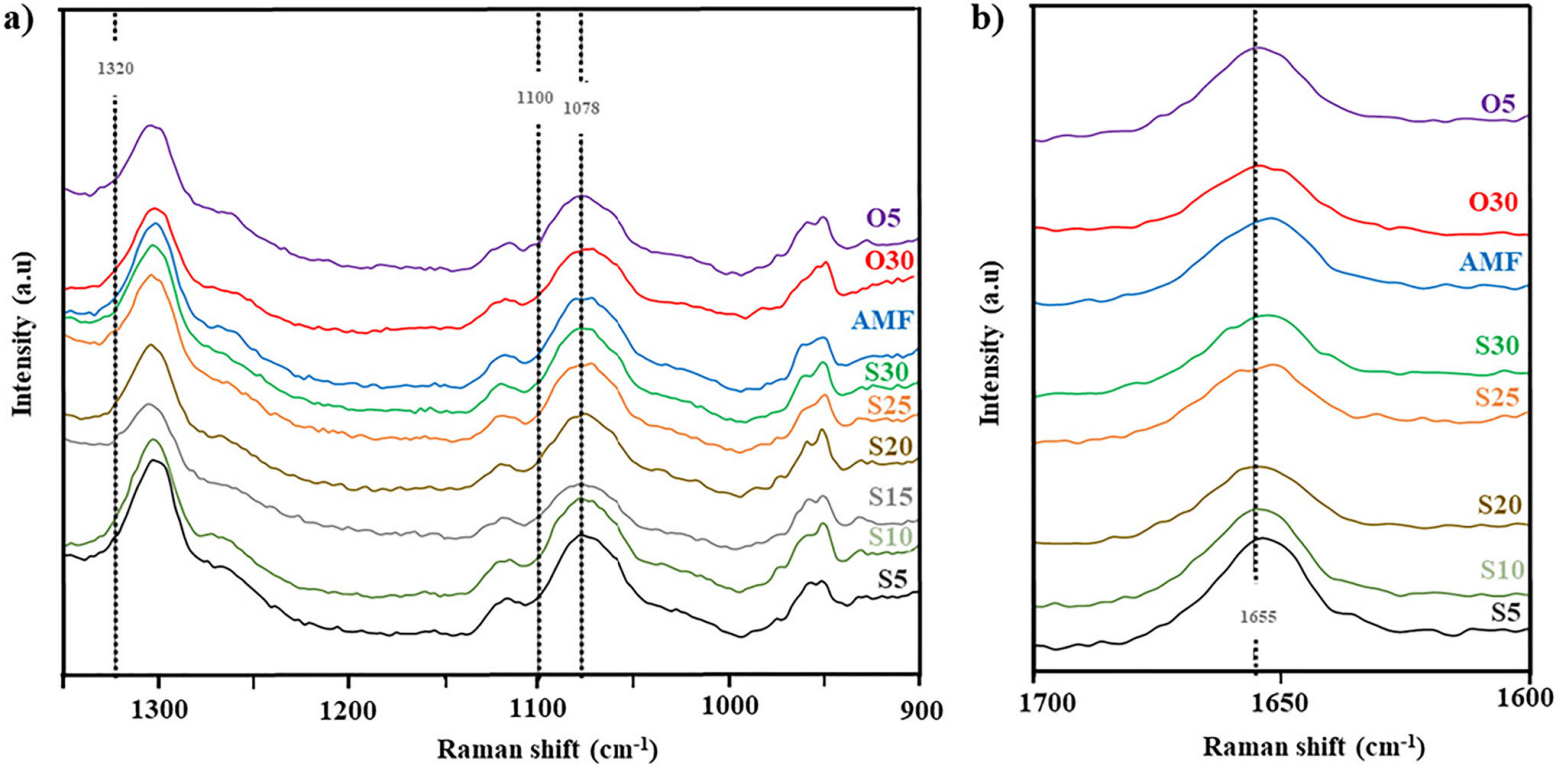
Raman spectra of anhydrous milk fat (AMF) and its dry‐fractionated samples obtained at different crystallization temperatures. (a) Spectral region from 900–1350 cm^−1^ and (b) spectral region from 1600–1700 cm^−1^.

The measurement temperature markedly affected the Raman spectra of lipids, particularly near their crystallization and melting transitions. At lower wavenumbers (900–1350 cm^−1^) (Figure 3a), several variations were observed that correlate with the degree of unsaturation in the AMF fractions, as previously identified by FTIR spectroscopy (Figure 2). A shift from 1005 cm^−1^, which appeared in AMF, to 995 cm^−1^ was detected in the O30, S5, and O5 samples. This band is associated with carbon backbone vibrations (Talari et al. 2015), indicating the unsaturation level and chain length of FAs. In addition, a further shift from 1078 to 1073 cm^−1^ was noted in the AMF, S30, and S25 fractions. This region corresponds to C─C stretching vibrations (Baeten et al. 1998; Czamara et al. 2015), suggesting the prevalence of LC‐SFAs. Bands in the 1300–1320 cm^−1^ region are primarily associated with CH_2_ twisting/deformation vibrations and are sensitive to lipid chain conformation, packing, and mobility. Differences observed in this spectral region among the AMF fractions suggest variations in molecular organization and chain mobility. Accordingly, the band at approximately 1320 cm^−1^ was interpreted as a qualitative indicator of differences in lipid chain packing and mobility rather than as a specific marker of any particular FA class. Furthermore, the peak at ∼1655 cm^−1^, attributed to C = C stretching vibrations in unsaturated fatty acids, (Gómez‐Mascaraque et al. 2021), was identified in all fractions according to GC‐based lipid profile. This peak shifted to lower wavenumbers at 1651 cm^−1^ in the AMF, S30, and S25 fractions, indicating reduced UFAs interactions (Figure 3b). In addition, this increased peak intensity was observed in O30, O5, S10, and S5 fractions, confirming their higher degree of unsaturation. Table 4 presents the peak shifts of the AMF fractions via Raman spectroscopy. Although the present analysis was primarily qualitative, further quantitative evaluation of Raman peak intensity ratios could provide deeper insight into the molecular organization and unsaturation levels of the AMF fractions.

**TABLE 4:** Raman shifts (cm^−1^) and corresponding vibrational assignments for anhydrous milk fat (AMF) and its solid (S) and liquid (O) fractions obtained at different crystallization temperatures. AMF fractions follow the convention used in Figure 1.

	AMF fractions									
	AMF	S30	S25	S20	S15	S10	S5	O30	O5	Peak assignment
**Raman Shifts (cm^−1^)**	1651	1651	1651	1655	1655	1655	1655	1655	1655	C═C groups in unsaturated fatty acids
	1320	1320	1320	1320	1320	—	—	—	—	CH_2_ twisting/deformation vibration; chain conformation, packing and mobility
	—	—	—	—	—	—	—	—	1100	ν (C─C)
	1073	1073	1073	1073	1078	1078	1078	1078	1078	ν (C─C)

#### 3.1.5 Thermal Analysis

**FIGURE 4:**
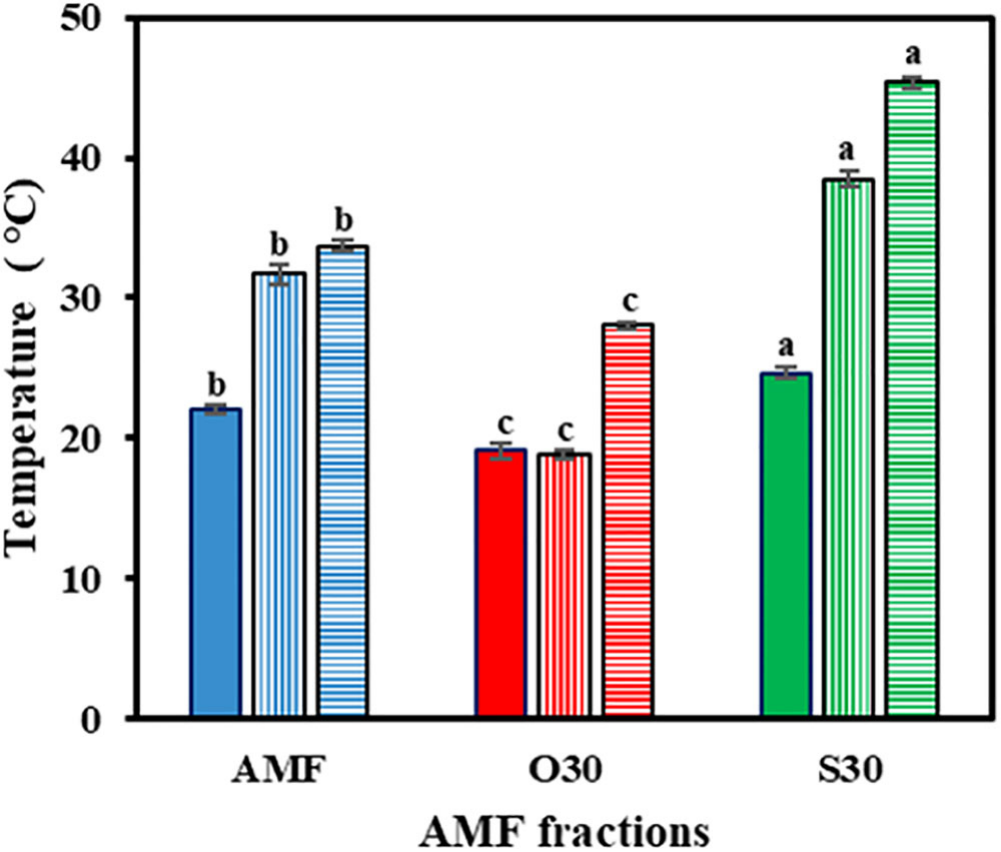
Thermal properties of anhydrous milk fat (AMF) and its dry fractions obtained at 30°C (O30: liquid fraction; S30: solid fraction). The solid bars represent the crystallization onset temperature (*T*_c_), whereas the vertically striped and horizontally striped bars correspond to the melting onset (*T*_m_) and melting offset temperatures, respectively. Different letters above each bar indicate statistically significant differences among treatments (*p* < 0.05).

Figure 4 summarizes the crystallization and melting transitions of AMF and its 30°C fractions as determined by μDSC analysis. The figure presents the crystallization onset (*T*_c_) temperatures of AMF, S30, and O30, as well as the melting onset (*T*_m_) and melting offset temperatures. Since crystallization behavior in MF systems can be influenced by thermal scan rates, identical cooling, and heating conditions were applied to all samples to ensure reliable comparison among the AMF fractions. Notably, the S30 exhibited higher crystallization temperatures than both AMF and O30, which can be attributed to its elevated proportion of LC‐SFAs. Regarding the melting behavior, S30 showed both higher melting onset and offset temperatures relative to AMF, whereas O30 displayed considerably lower melting transitions. The ∼20°C difference between the melting temperatures of S30 and O30 may be attributed to the variation in the saturation level (SFAs/UFAs). Moreover, the melting offset of the S30 fraction was significantly higher than that of AMF (*p* < 0.05), underlying the enhanced thermal stability of the S30 fraction. In contrast, the O30 fraction exhibited a melting offset around 28°C (Figure 4), which was expected given that the O30 fraction was fully liquid at 30°C. The higher crystallization temperatures observed for the solid fractions, particularly S30, may have promoted earlier crystal nucleation and faster network formation within the oil phase, contributing to the enhanced structuring efficiency and stronger rheological behavior of the corresponding oleogels. Similar results on the crystallization and melting temperatures of fractions have been reported in other studies (Li et al. 2017; Y. Wang et al. 2019). Li et al. (2017) reported a crystallization temperature of 22.2°C and a melting offset temperature at 37.7°C at cooling and heating rates of 2 and 10°C/min, respectively. Similarly, Y. Wang et al. (2019) studied multi‐stage MF fractions, observing a crystallization onset at 25.7°C, a melting point at 38.2°C and a melting offset at 44.5°C for the solid fraction obtained at 30°C.

#### 3.1.6 Rheological Behavior

**FIGURE 5:**
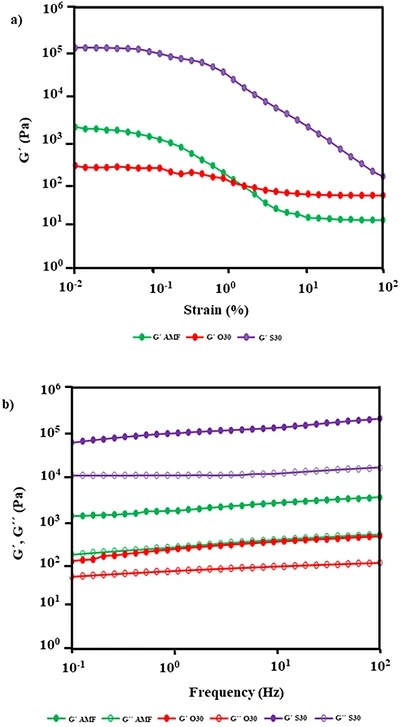
Rheological properties of anhydrous milk fat (AMF) and its dry fractions obtained at 30°C (O30: liquid fraction; S30: solid fraction). (a) Strain sweep tests showing the evolution of the storage modulus (*G*′) with increasing strain and b) frequency sweep tests showing the storage modulus (*G*′) and loss modulus (*G*″) as a function of frequency (0.1–100 Hz). All samples were stored for 7 days before the measurement. The test was performed at 20°C.

**FIGURE 6:**
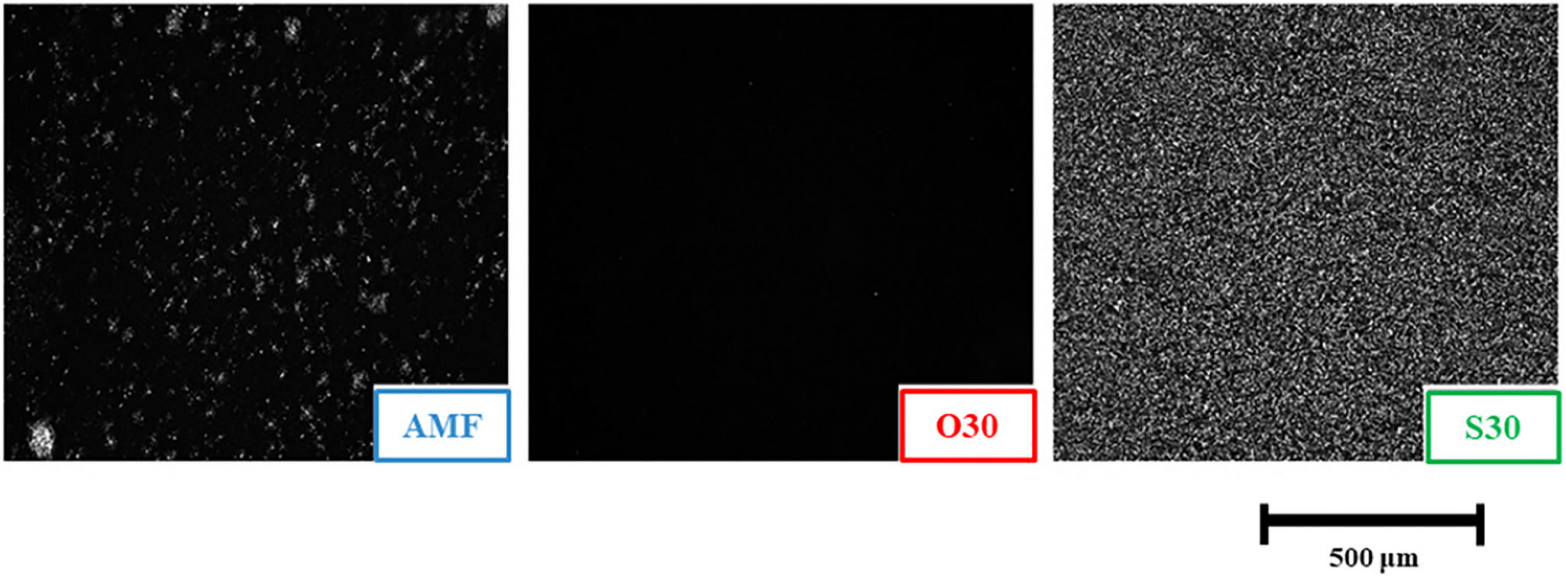
Polarized light microscopy images of anhydrous milk fat (AMF) and its dry‐fractionated samples obtained at 30°C, including the liquid fraction (O30) and the solid fraction (S30). Scale bar: 500 µm.

Figure 5 presents the rheological behavior of AMF and its fractions at 30°C (S30 and O30). The strain sweep analysis (Figure 5a) revealed significant differences in the network strength among the samples. The S30 fraction exhibited the highest *G*′ values, reaching approximately 10^5^ Pa within the LVR, greatly exceeding those of both AMF and O30. This indicates the formation of a dense and well‐structured crystalline network, consistent with the microstructural observations in Figure 6. Furthermore, *G*′ values of S30 fractions remained at elevated levels across a broad strain range, demonstrating high resistance to deformation. On the contrary, AMF exhibited a more rapid decline in *G*′ beyond the LVR, while the O30 fraction showed weak gel‐like behavior, indicating limited crystallinity. Frequency sweep results (Figure 5b) show that the AMF, S30, and O30 fractions exhibited solid‐like behavior at 20°C (*G*′ > *G*″), in agreement with their melting offset temperatures above 28°C (Figure 4). In addition, the *G′* values at 1 Hz differed significantly among samples (Table 5), with S30 exhibiting the highest value, confirming the formation of a robust crystalline network. These rheological differences in elasticity were attributed to their compositional differences, particularly the SFA‐UFA ratio (Table 2), which significantly affected the crystallization behavior and the resulting microstructure of the fractions (Prodromidis et al. 2024; Y. Wang et al. 2023). In addition, the flow point of the S30 fraction was significantly lower than that of AMF (Table 5), demonstrating that once the deformation initiated, the S30 network underwent a more abrupt structural breakdown. This behavior is indicative of a more brittle‐like behavior and densely packed crystalline microstructure, further supporting the thermal and microscopy findings.

**TABLE 5:** Rheological properties of anhydrous milk fat (AMF) and its fractions obtained by dry fractionation at 30°C (O30: liquid fraction and S30: solid fraction).

Rheological properties	AMF fractions		
	ΑMF	O30	S30
***G*′ (Pa) at 1 Hz**	1500 ± 300^b^	199 ± 52^c^	90,400 ± 10,500^a^
**Tan*δ* at 1 Hz**	0.20 ± 0.05^b^	0.30 ± 0.02^a^	0.10 ± 0.04^c^
**Yield stress (Pa)**	1.54 ± 0.30^b^	—	4.03 ± 0.70^a^
**Flow point (Pa)**	2.20 ± 0.40^a^	—	1.10 ± 0.20^b^

*Note*: Values are expressed as means ± standard deviations. Different superscript letters within the same row indicate statistically significant differences among treatments (*p* < 0.05).

#### 3.1.7 Microstructure of AMF Fractions

The microstructure of AMF and its fractions crystallized at 30°C was examined using PLM. As shown in Figure 6, unfractionated AMF displayed a characteristic morphology of large crystalline aggregates, consistent with previous studies (Viriato et al. 2018; Małkowska et al. 2021; Mohan et al. 2021). In contrast, the S30 fraction exhibited a higher density of smaller, uniformly distributed crystals, indicative of a more organized and cohesive crystalline network. Such finely structured networks are typically associated with increased hardness and enhanced structural integrity. The O30 fraction, however, showed only sparse crystalline domains that were insufficient to form a continuous and stable fat crystal network, reflecting its limited ability to provide structural reinforcement. This microstructure agrees with its lower SFAs content (Table 2) and weaker rheological properties (Figure 5). Overall, the microstructural differences among AMF, S30, and O30 support the thermal and rheological findings, demonstrating that fractionation at 30°C produced a solid fraction (S30) with markedly improved crystallization capacity.

### 3.2 Characterization of Oleogels Prepared With AMF Fractions

#### 3.2.1 Oleogelation Potential of AMF Fractions

**FIGURE 7:**
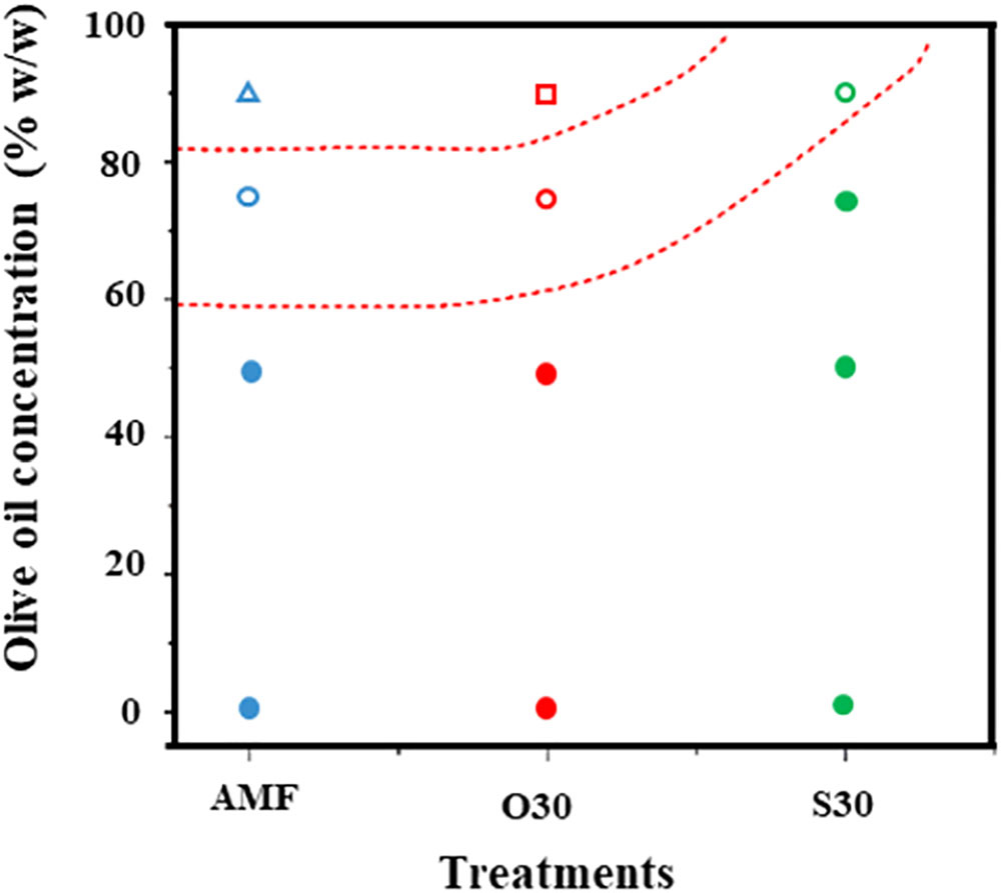
Oleogelation potential of anhydrous milk fat (AMF) and its dry‐fractionated samples obtained at 30°C (O30: liquid fraction; S30: solid fraction). The plot shows the structuring behavior of each fat system at different olive oil concentrations (% w/w). Symbols indicate the type of physical behavior observed: ● free‐standing gel, °turbid high‐viscosity system, □ transparent low‐viscosity system, and △ phase separation. The dashed red lines illustrate the transition boundaries between the different structural states.

**FIGURE 8:**
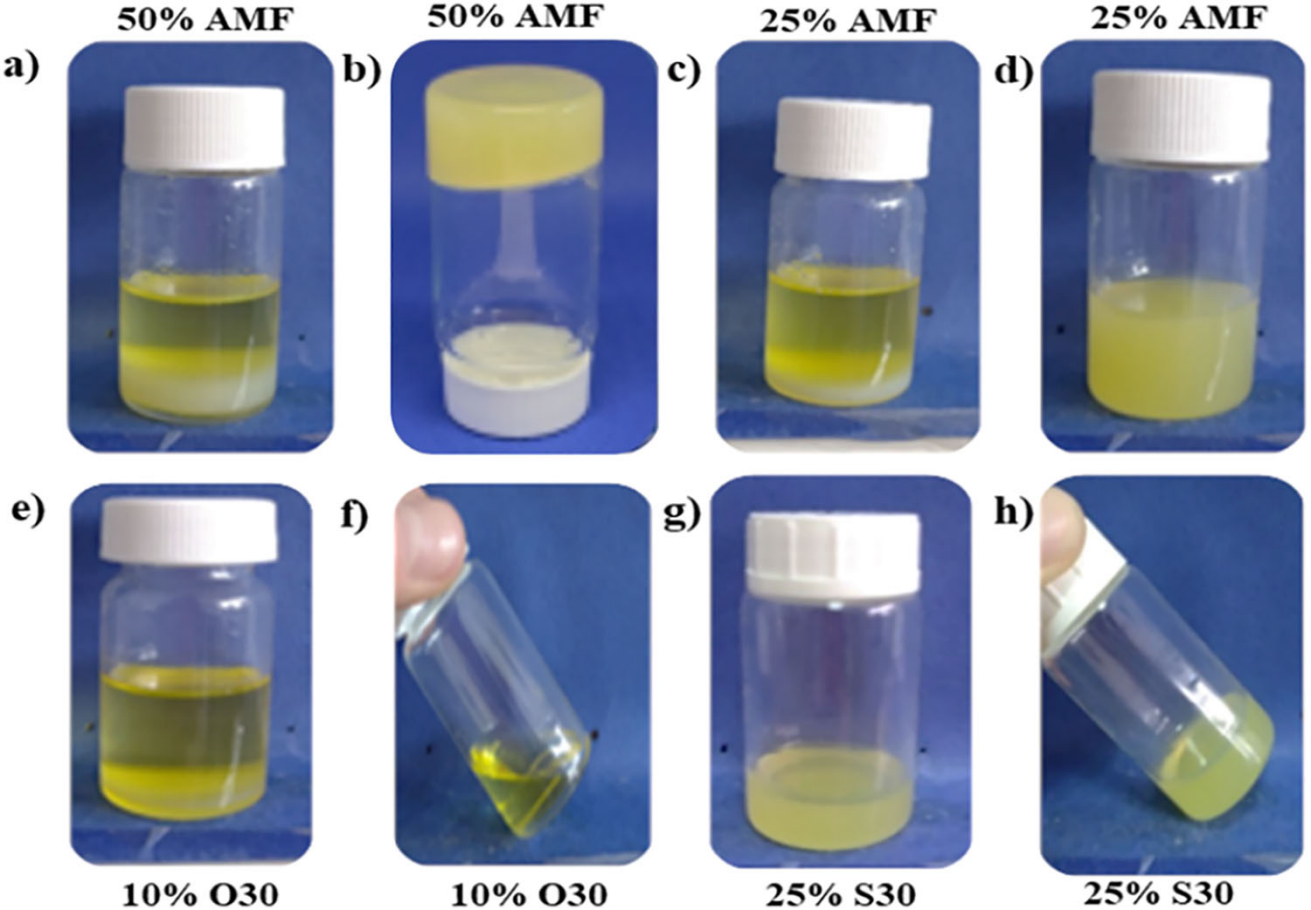
Macroscopic appearance of anhydrous milk fat (AMF) and its fractionated samples in olive oil blends stored at 20°C for 7 days. Images illustrate the visual stability and phase behavior of: (a,b) 50% AMF, (c,d) 25% AMF, (e,f) 10% O30 (low–melting liquid fraction obtained at 30°C), and (g,h) 25% S30 (high–melting solid fraction obtained at 30°C).

The oleogelation potential of AMF and its dry‐fractionated derivatives was evaluated at various structurant concentrations in olive oil (Figure 7). Unfractionated AMF produced a self‐standing gel only at the 50:50 ratio with olive oil. At the same time, blends containing 25% AMF formed a turbid, high viscosity system, and those containing 10% AMF remained transparent and fluid. Stable, self‐standing gels were obtained only after cooling to low temperatures (e.g., around 0°C) (Figure 8), reflecting the broad crystallization temperature range of AMF and its fraction (−40°C up to 40°C) (Si et al. 2023). Without such cooling, phase separation occurred (Figure 8a,c), confirming that crystallization is essential for network formation. At low AMF concentrations, the absence of a sufficiently dense crystal network resulted in a homogeneous thick liquid (Figure 8d). These observations align with earlier studies showing that AMF vegetable oil blends require ≥ 50% AMF to form structured systems (Gómez‐Mascaraque et al. 2021). While the oleogelation behavior of unfractionated AMF has previously been reported, the present study focuses on how dry fractionation modifies the physicochemical and structuring properties of AMF fractions.

According to Figure 7, the S30 fraction demonstrated the highest oleogelation potential among all treatments. In particular, the minimum concentration required for self‐standing gel formation was lower for S30 (25%) compared to AMF and O30, which required at least 50% structurant concentration to form stable oleogels. Therefore, based on their distinct thermal and physicochemical characteristics, the AMF, S30, and O30 fractions were selected for oleogel preparation. In more detail, the S30 fraction produced a self‐standing gel at a 50:50 ratio and notably retained its structuring ability even at much lower concentrations. For example, at 25% S30 fraction, stable, self‐standing gels were achieved (Figures 7 and 8h), while at 10% the formation of thick cohesive gels was obtained, indicating that a crystalline network developed despite the reduced structurant level. In contrast, the O30 fraction formed a self‐standing gel only at the 50:50 ratio but failed to structure olive oil at lower concentrations. At 25% O30 fraction, the blend appeared as a homogenous viscous liquid, while at 10% O30, the mixture remained fully fluid and transparent, with no evidence of network formation (Figure 8e). The superior gelling efficiency of S30 compared to other fractions was attributed to its elevated content of SFAs, primarily palmitic and stearic acids, which promote crystallinity and lead to the formation of dense and robust networks at low temperatures. Intermediate fractions, such as the S25 and S20, also formed homogeneous viscous gels at a 25:75 ratio after cooling to 0°C (data not shown), whereas the lower melting fractions failed to yield structured systems.

#### 3.2.2 Thermophysical Properties

The thermal properties of the produced oleogels using AMF and its fractions were examined using μDSC (Table 6). Significant differences were detected between oleogels structured with either 25% AMF or 25% S30 fraction. The AMF‐based oleogels exhibited significantly lower crystallization onset and melting transition temperatures compared to the 25% S30 oleogels (*p* < 0.05). The low crystallization point of AMF oleogel (6.2 ± 0.2°C) indicates that a limited number of crystals formed upon cooling to 0°C, which explains its inability to produce a stable self‐standing gel. In contrast, the 25% S30 oleogel displayed a significantly higher onset of crystallization (15.6 ± 0.2°C), confirming that a larger proportion of its high‐melting FAs crystallized well before reaching 0°C. This early crystallization allowed the development of a continuous crystalline and self‐standing network. Similarly, the higher melting onset and offset temperatures of the S30 oleogels further confirmed their improved thermal stability and more robust crystalline network relative to the AMF‐based oleogels.

**TABLE 6:** Crystallization onset and melting transitions (onset and offset temperatures) of oleogels structured with anhydrous milk fat (AMF) and its high‐melting solid fraction (S30), as determined by μDSC analysis.

Oleogels	Crystallization onset, °C	Melting point (onset), °C	Melting point (offset), °C
**25% AMF**	6.2 ± 0.2^a^	19.4 ± 0.4^a^	29.8 ± 0.3^a^
**25% S30**	15.6 ± 0.2^b^	24.5 ± 0.3^b^	35.3 ± 0.5^b^

*Note*: Values are reported as means ± standard deviations. Different superscript letters within the same column indicate statistically significant differences between oleogels (*p* < 0.05).

#### 3.2.3 Microstructure

**FIGURE 9:**
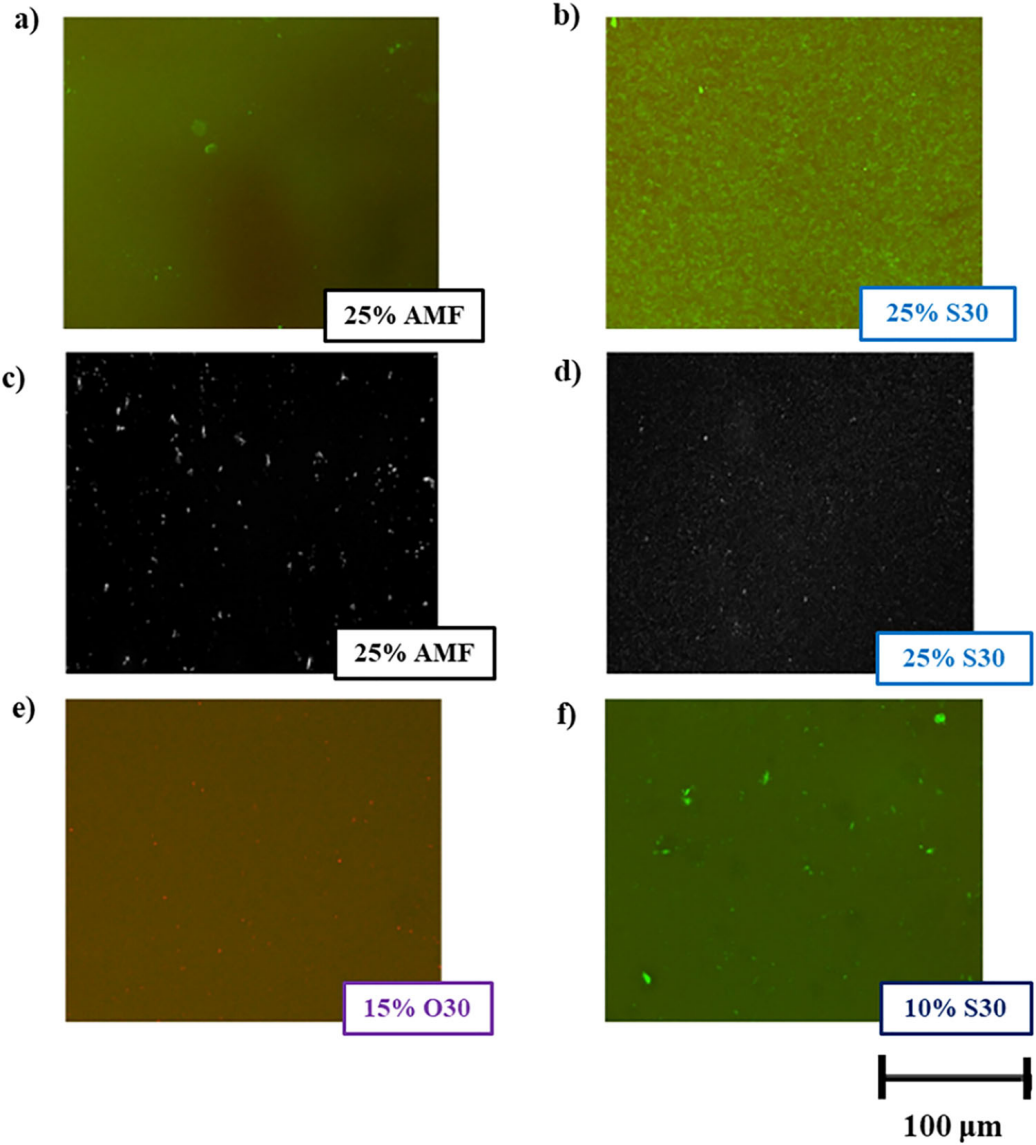
Confocal laser scanning microscopy (CLSM) micrographs (a, b, e, f) and polarized light microscopy (PLM) images (c, d) of olive oil oleogels formulated with anhydrous milk fat (AMF), the high‐melting solid fraction (S30) and the low‐melting liquid fraction (O30). In the CLSM images, the lipid phase appears in red and the crystalline structures in green. Scale bar: 100 µm.

The microstructure of the oleogels was assessed using CLSM and PLM. The CLSM images (Figure 9a,b,e,f) clearly show the distribution of the crystalline phase (green) within the continuous olive oil phase (red). Oleogels structured with AMF exhibited large, irregular crystalline aggregates of TAG crystals (Figure 9a,c), characteristic of spherulitic structures associated with β‐polymorph crystallization (Viriato et al. 2018; Małkowska et al. 2021; Mohan et al. 2021). These heterogeneous crystal clusters correspond with the moderate oleogelation ability of AMF.

In contrast, oleogels prepared with 25% S30 displayed a much finer and more homogeneous crystalline network (Figure 9b,d). The crystals were smaller and more uniformly distributed throughout the olive oil phase, indicating the improved crystallization efficiency and enhanced structuring ability of this fraction. The CLSM image shown in Figure 9e further confirms the formation of a dense crystalline network capable of effectively immobilizing the liquid oil phase. Even at 10% S30 in olive oil (Figure 9f), the microstructure remained composed of small, well‐dispersed crystallites, consistent with its macroscopic appearance as a thick and cloudy system.

#### 3.2.4 Rheological Behavior

**FIGURE 10:**
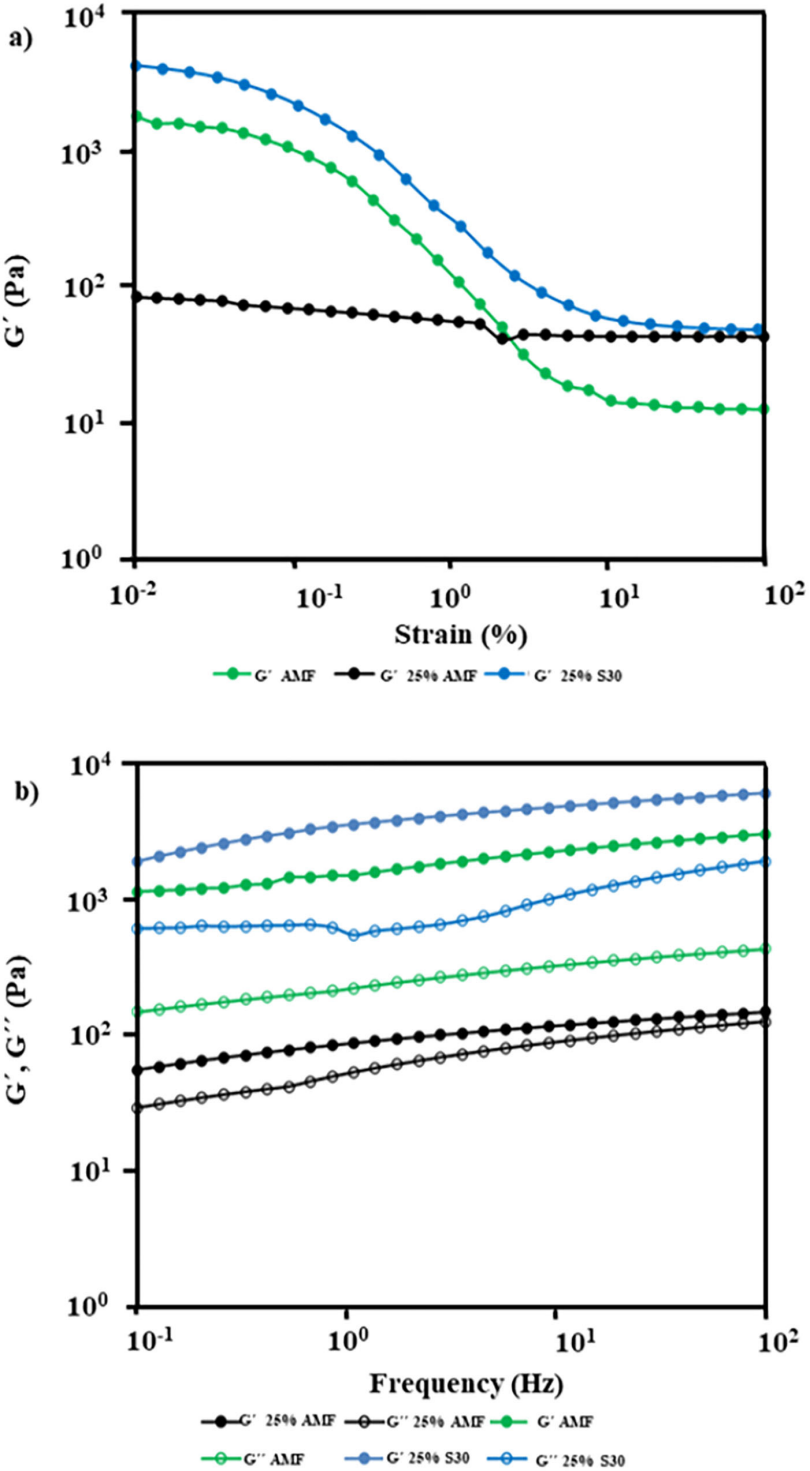
Small‐amplitude oscillatory shear (SAOS) rheological behavior of anhydrous milk fat (AMF) and AMF‐based systems formulated either as AMF/olive oil blends or as oleogels containing 25% (w/w) of the high‐melting solid fraction obtained at 30°C (S30). (a) Strain sweep test illustrating the evolution of the storage modulus (G′) with increasing strain and (b) frequency sweep test showing the storage modulus (G′) and loss modulus (G″) as a function of frequency (0.1–100 Hz). All measurements were performed at 20°C, 7 days after preparation.

Small‐strain oscillation tests revealed significant differences in the mechanical properties of the oleogels. The strain sweeps data (Figure 10a) confirmed the superior structural integrity of the S30 oleogels. Although all oleogels exhibited *G′* > *G*″ within the LVR, the S30 oleogel maintained the highest *G′* values over a broader strain range. Interestingly, the S30 oleogel showed a significantly lower flow point (1.10 ± 0.30 Pa) compared to pure AMF (2.21 ± 0.40 Pa), suggesting that once deformation initiates, the S30 network undergoes a more abrupt structural breakdown. Such behavior is characteristic of densely packed, brittle crystalline networks and aligns well with the observed microstructure (Figure 9). Conversely, the oleogel containing 25% AMF lacked a measurable yield stress or flow point, reflecting the absence of a continuous crystalline network and confirming its weak viscoelastic behavior. The frequency sweep results (Figure 10b) further supported the differences in network rigidity among the oleogels. The oleogel containing 25% AMF exhibited the weakest elastic response, with *G*′ values significantly lower than those of both pure AMF and the 25% S30 oleogel (Table 7). The close values of *G*′ and *G*″ throughout the frequency range indicate a weakly developed structure with predominantly viscous behavior, consistent with its fluid‐like macroscopic appearance. In contrast, the 25% S30 oleogel demonstrated a stronger elastic structure. Its *G*′ at 1 Hz (3540 ± 630 Pa) was more than double that of unfractionated AMF (1500 ± 300 Pa) and nearly 40‐fold higher than the 25% AMF blend (87 ± 35 Pa). These results demonstrate that even at a relatively low concentration, the S30 fraction acts as an efficient structurant, forming a dense crystalline network that imparts solid‐like behavior. This is further supported by its low tan*δ* values, indicative of clear elastic dominance (*G*′ >> *G*″).

**TABLE 7:** Rheological parameters of anhydrous milk fat (AMF) and oleogels formulated using either 25% (w/w) of the high‐melting solid fraction obtained at 30°C (S30) or blends of AMF with olive oil. Measurements were performed at 20°C, 7 days after sample preparation.

Rheological properties	Oleogels		
	ΑMF	25% S30	25% AMF
***G*′ (Pa) at 1 Hz**	1500 ± 300^b^	3540 ± 630^a^	87.10 ± 35.00^c^
**Tanδ at 1 Hz**	0.20 ± 0.10^b^	0.15 ± 0.14^c^	0.61 ± 0.20^a^
**Yield stress (Pa)**	1.54 ± 0.30^b^	2.03 ± 0.20^a^	—
**Flow point (Pa)**	2.21 ± 0.40^a^	1.10 ± 0.30^b^	—

*Note*: Values represent means ± standard deviations. Different superscript letters within the same row indicate statistically significant differences among oleogels (*p* < 0.05).

## 4 Conclusions

This study demonstrated that AMF predominantly consists of high‐melting‐point components and that dry fractionation effectively separates AMF into fractions with distinct physicochemical characteristics. Solid fractions obtained at high temperatures, particularly S30, contained a high level of SFAs, as confirmed by GC‐FID, FT‐IR and Raman spectroscopy results. The S30 fraction also exhibited significantly higher crystallization onset and melting point temperatures than AMF, supporting the potential of oleogelation. The ability of AMF and its fraction to structure olive oil depended strongly on their crystallization behavior during cooling. Unfractionated AMF produced a self‐standing oleogel only at a 50:50 ratio, whereas lower ratios resulted in viscous or phase‐separated systems. However, the S30 fraction demonstrated markedly superior structuring ability, forming self‐standing oleogels at a 25:75 ratio and generating robust crystalline networks even at reduced concentrations. The corresponding liquid fraction, O30, exhibited limited crystallization and thus minimal oleogelation capacity, acting as a structurant only at the highest concentration tested. Notably, the 25% S30 oleogel demonstrated a strong elastic response, highlighting the high structuring efficiency of the S30 fraction, which was able to form a robust crystalline network even at relatively low concentrations in oil. However, these systems exhibited increased brittle‐like behavior due to their denser microstructure, consistent with thermal, microscopic, and rheological findings. These results demonstrate that dry‐fractionated AMF, particularly the S30 fraction, can serve as an efficient, natural oleogelator for structuring liquid oils into stable oleogels. It should be noted that S30 itself is not a reduced‐SFA fraction; rather, it is enriched in saturated fatty acids but exhibits high structuring efficiency, enabling the formation of stable oleogels at relatively low concentrations. Consequently, the resulting oleogel, containing 25% S30 and 75% olive oil, may provide a structured lipid system with a lower overall saturated fatty acid content than formulations based entirely on AMF or other conventional solid fats. Overall, these findings highlight the potential of dry‐fractionated AMF as a food‐grade, solvent‐free structurant for the development of alternative structured lipid systems with tailored functionality. Further studies are required to evaluate the storage stability, oil binding capacity, and oxidative stability of these oleogel systems under practical application conditions, as well as their performance in real food matrices such as bakery and confectionery products.

## Author Contributions

**Konstantina Zampouni**: writing – review and editing, visualization, formal analysis, data curation. **Prodromos Prodromidis**: visualization, writing – original draft, formal analysis, data curation, investigation. **Elefterios G. Andriotis**: writing – review and editing. **Eugenios Katsanidis**: writing – review and editing, validation. **Thomas Moschakis**: conceptualization, funding acquisition, methodology, visualization, writing – review and editing, project administration, supervision, resources.

## Conflicts of Interest

The authors declare no conflicts of interest.

## Data Availability

The data presented in this study are available on request from the corresponding author.
